# 3-Hydroxyolean-12-en-27-oic Acids Inhibit RANKL-Induced Osteoclastogenesis in Vitro and Inflammation-Induced Bone Loss in Vivo

**DOI:** 10.3390/ijms21155240

**Published:** 2020-07-23

**Authors:** Wonyoung Seo, Suhyun Lee, Phuong Thao Tran, Thi Quynh-Mai Ngo, Okwha Kim, Thanh Huong Le, Nguyen Hai Dang, Cheol Hwangbo, Byung Sun Min, Jeong-Hyung Lee

**Affiliations:** 1 Department of Biochemistry, Kangwon National University, Chuncheon, Gangwon-Do 24341, Korea; swy4262@naver.com (W.S.); dltngus328@gmail.com (S.L.); helytran@gmail.com (P.T.T.); kah3173@kangwon.ac.kr (O.K.); 2 Kangwon Institute of Inclusive Technology, Kangwon National University, Chuncheon, Gangwon-Do 24341, Korea; 3 College of Pharmacy, Catholic University of Daegu, Gyeongbuk 38430, Korea; ngoquynhmai2011@gmail.com (T.Q.-M.N.); bsmin@cu.ac.kr (B.S.M.); 4 University of Science and Technology of Hanoi, Vietnam Academy of Science and Technology, 18 Hoang Quoc Viet, Caugiay, Hanoi 100000, Vietnam; le-thanh.huong@usth.edu.vn (T.H.L.); haidangnguyen@gmail.com (N.H.D.); 5 Division of Applied Life Science (BK21 Plus), PMBBRC, Division of Life Science, College of Natural Sciences, Gyeongsang National University, Jinju 52828, Korea; chwangbo@gnu.ac.kr

**Keywords:** 3-hydroxyolean-12-en-27-oic acid, osteoclastogenesis, RANKL, c-Fos, NFATc1

## Abstract

Olean-12-en-27-oic acids possess a variety of pharmacological effects. However, their effects and underlying mechanisms on osteoclastogenesis remain unclear. This study aimed to investigate the anti-osteoclastogenic effects of five olean-12-en-27-oic acid derivatives including 3α,23-isopropylidenedioxyolean-12-en-27-oic acid (AR-1), 3-oxoolean-12-en-27-oic acid (AR-2), 3α-hydroxyolean-12-en-27-oic acid (AR-3), 23-hydroxy-3-oxoolean-12-en-27-oic acid (AR-4), and aceriphyllic acid A (AR-5). Among the five olean-12-en-27-oic acid derivatives, 3-hydroxyolean-12-en-27-oic acid derivatives, AR-3 and AR-5, significantly inhibited receptor activator of nuclear factor-κB ligand (RANKL)-induced mature osteoclast formation by reducing the number of tartrate-resistant acid phosphatase (TRAP)-positive osteoclasts, F–actin ring formation, and mineral resorption activity. AR-3 and AR-5 decreased RANKL-induced expression levels of osteoclast-specific marker genes such as c-Src, TRAP, and cathepsin K (CtsK) as well as c-Fos and nuclear factor of activated T cells cytoplasmic 1 (NFATc1). Mice treated with either AR-3 or AR-5 showed significant protection of the mice from lipopolysaccharide (LPS)-induced bone destruction and osteoclast formation. In particular, AR-5 suppressed RANKL-induced phosphorylation of JNK and ERK mitogen-activated protein kinases (MAPKs). The results suggest that AR-3 and AR-5 attenuate osteoclast formation in vitro and in vivo by suppressing RANKL-mediated MAPKs and NFATc1 signaling pathways and could potentially be lead compounds for the prevention or treatment of osteolytic bone diseases.

## 1. Introduction

Bone maintains its structure and function through coordinating the activity of osteoclasts and osteoblasts, a process known as bone remodeling [1,2]. Osteoclasts are derived from hematopoietic progenitors in the bone marrow and are formed through the differentiation of mononucleated osteoclast precursor cells. Osteoclasts are the sole cells in the body that are capable of bone resorption [3,4], and can be embedded on the bone surface and function through secretion of several enzymes to degrade the bone matrix [4,5]. Excessive osteoclast activity causes bone loss, which leads to numerous pathological bone diseases such as rheumatoid arthritis, tumor bone metastases, osteoporosis, and Paget’s disease [6,7].

The differentiation of osteoclast is regulated by two crucial cytokines, macrophage colony-stimulating factor (M-CSF) and receptor activator of nuclear factor-κB ligand (RANKL) [8,9,10,11,12]. M-CSF mediates the survival and proliferation of early macrophage/osteoclast precursors and induces the receptor activator of nuclear factor-κB (RANK) expression. On the other hand, RANKL is essential for osteoclastogenesis through the interaction with its receptor RANK on the cell membrane of these precursors. The binding of RANKL to RANK results in the recruitment of TNF-receptor-associated factor 6 (TRAF6) and activation of downstream signaling molecules such as mitogen-activated protein kinases (MAPKs) and nuclear factor-κB (NF-κB), which later activate c-Fos and nuclear factor of activated T cells cytoplasmic 1 (NFATc1) [13,14,15,16]. The activation of NFATc1 directly controls the expression of various osteoclastic genes including matrix metallopeptidase-9 (MMP-9), osteoclast-associated receptor (OSCAR), TRAP, and dendritic cell–specific transmembrane protein (DC-STAMP), critical for osteoclast formation and function [5,17].

Natural products have been utilized as an excellent source for new drug discovery [18]. Natural products can either be directly utilized as drugs to treat diseases in humans, or at least serve as a valuable lead for drug discovery [18,19,20]. Triterpenes are one of the most interesting classes of natural products [21]. Pentacyclic triterpenes have received considerable attention due to the benefits on human health with some being marketed as dietary supplements or therapeutic agents around the world [22]. In our search for new types of chemical entities from natural products that might prevent osteoclast formation, we discovered that two 3-hydroxyolean-12-en-27-oic acid derivatives, β-peltoboykinolic acid (AR-3) and aceriphyllic acid A (AR-5), derived from *Aceriphyllum rossi* Engler (Saxifragaceae) suppressed RANKL-induced osteoclast formation. 3-Hydroxyolean-12-en-28-oic acid derivatives including oleanolic acid and maslinic acid, exert anti-osteoclastogenic effects in vitro and in vivo [23,24]. However, the effects and underlying mechanisms of oleanane triterpenes with a carboxyl group at C-27 on osteoclast formation and function are yet to be studied. Therefore, the aim of this study was to investigate the anti-osteoclastogenic effects of 3-hydroxyolean-12-en-27-oic acid derivatives in vitro and in vivo.

## 2. Results

### 2.1. AR-3 and AR-5 Inhibits RANKL-Induced Osteoclast Formation in RAW264.7 Cells

**Figure 1:**
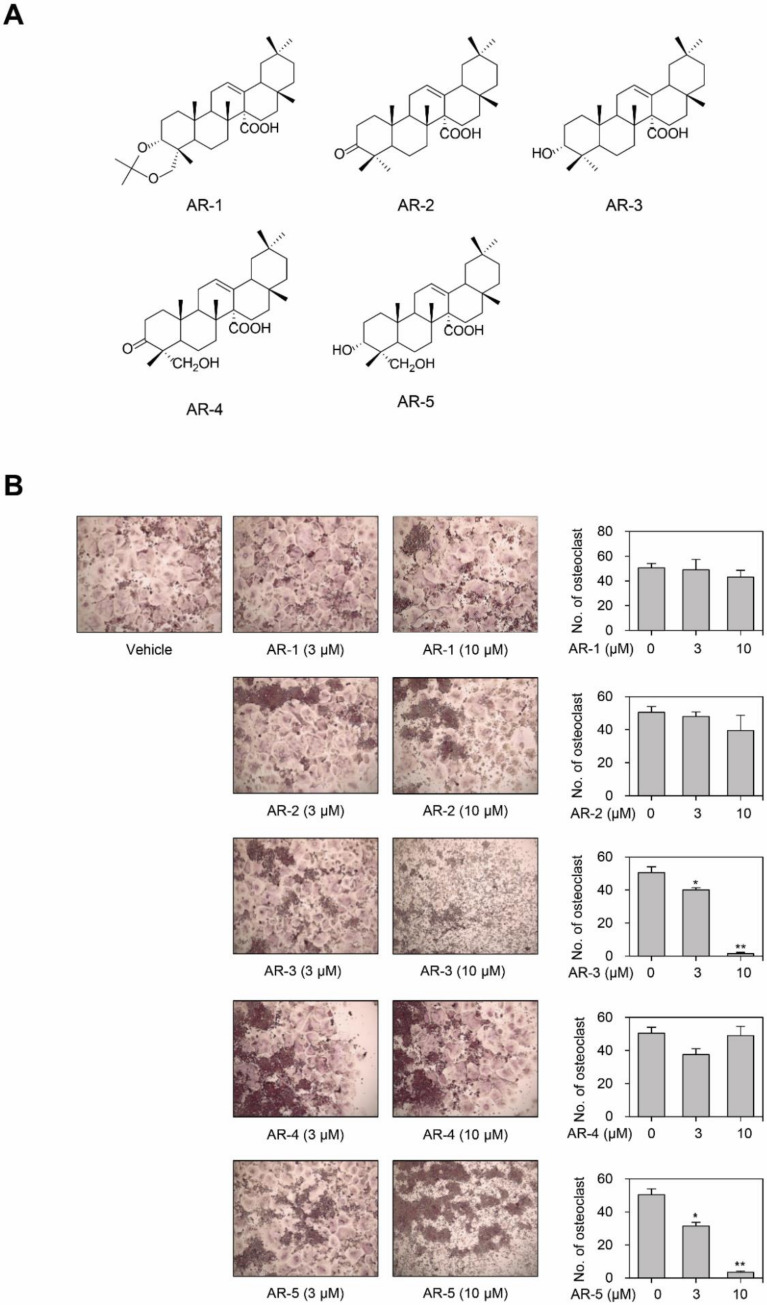
Olean-12-en-27-oic acid derivatives inhibit RANKL-induced osteoclastogenesis. (**A**) Chemical structures of olean-12-en-27-oic acid derivatives isolated from *A. rossi*. (**B**) Effect of olean-12-en-27-oic acid derivatives on RANKL-induced osteoclastogenesis. RAW264.7 cells were cultured into 96 well-plates under stimulation of RANKL with or without different concentrations of the indicated compound for four days, then the cells were stained for TRAP. The quantities of TRAP-positive multinucleated (> 5 nuclei) osteoclasts were determined following image capture. Data are presented as the mean ± SE (* *p* < 0.05 and ** *p* < 0.01, versus vehicle-treated control; *n* = 3).

In order to identify novel anti-osteoclastogenic compounds from natural products, we evaluated the anti-osteoclastogenic effects of five olean-12-en-27-oic acid derivatives isolated from *A. rossi* in RAW264.7 cells (Figure 1A). Stimulation with RANKL efficiently differentiated RAW264.7 cells into multinuclear TRAP-positive osteoclasts. There was no significant decrease in the number of TRAP-positive multinucleated osteoclasts in RAW264.7 cells for AR-1, AR-2, and AR-4. However, the treatment of RAW264.7 cells with either AR-3 or AR-5 decreased the number of TRAP-positive multinucleated osteoclasts in a concentration-dependent manner (Figure 1B).

### 2.2. AR-3 and AR-5 Inhibit RANKL-Induced Osteoclast Formation in Mouse BMMs

**Figure 2:**
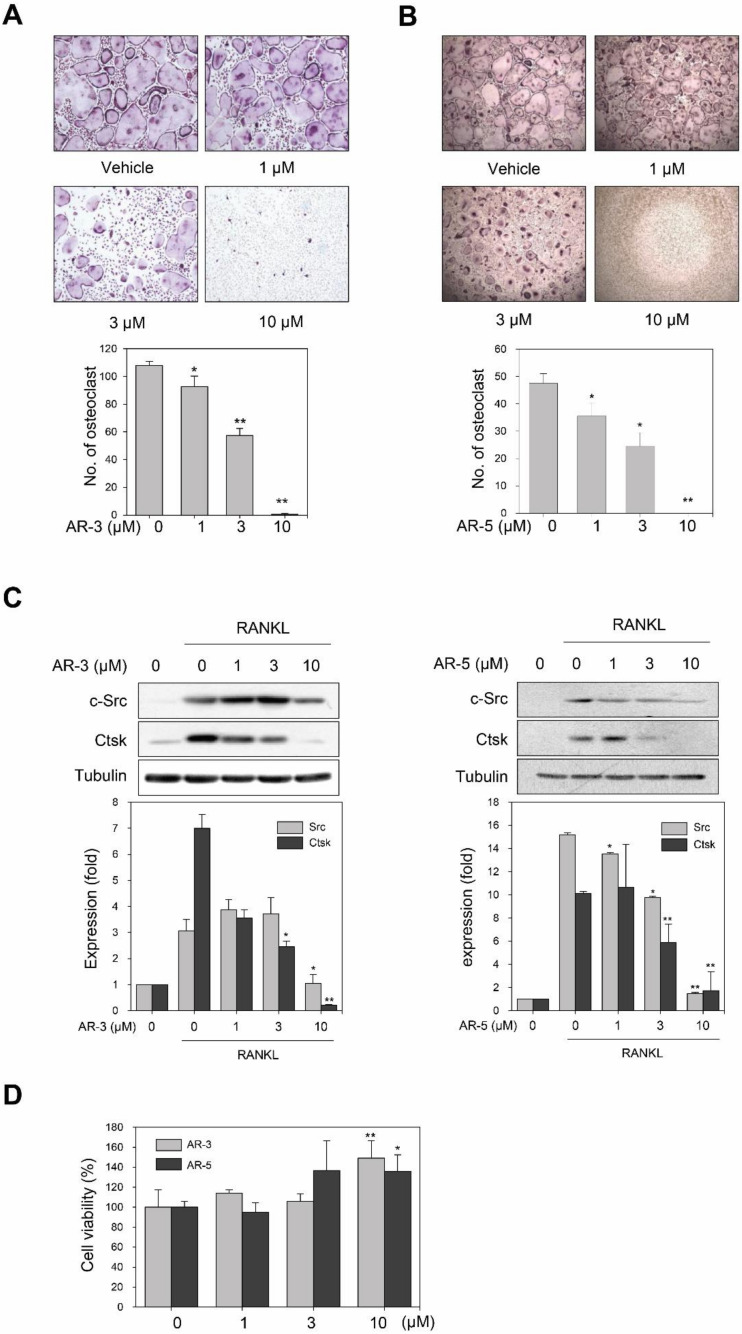
AR-3 and AR-5 suppress RANKL-induced osteoclastogenesis in BMMs. (**A**,**B**) BMMs were treated with the indicated concentrations of AR-3 (**A**) or AR-5 (B), and then stimulated with M-CSF and RANKL for seven days. The cells were stained for TRAP. The number of TRAP-positive osteoclasts (>5 nuclei) was determined following image capture. Data are presented as the mean ± SE (* *p* < 0.05 and ** *p* < 0.01, versus vehicle-treated control; *n* = 3). (**C**) BMMs were treated with the indicated concentrations of AR-3 (left panel) or AR-5 (right panel), and then stimulated with RANKL for four days. Total lysates were prepared and the expression levels of c-Src and CtsK were determined by western blotting. Graphs represent densitometry analyses normalized to tubulin (* *p* < 0.05 and ** *p* < 0.01, versus vehicle-treated control; *n* = 3). (**D**) BMMs were incubated with M-CSF in the presence of the indicated concentrations of AR-3 or AR-5 for four days. Cell viability was measured using the MTT assay. Data are presented as the mean ± SE (* *p* < 0.05 and ** *p* < 0.01, versus vehicle-treated control; *n* = 3).

To further verify that AR-3 and AR-5 could inhibit RANKL-induced osteoclast formation, we determined whether AR-3 and AR-5 could suppress RANKL-induced osteoclast in primary cultured bone marrow-derived macrophages (BMMs). Stimulation of mouse BMMs with M-CSF and RANKL induced osteoclast differentiation from the precursors. However, the treatment of BMMs with AR-3 or AR-5 suppressed the formation of TRAP-positive multinucleated osteoclasts in a concentration-dependent manner (Figure 2A,B). The IC_50_ values of AR-3 and AR-5 were 2.9 ± 0.3 and 2.6 ± 0.2 µM, respectively. As a positive compound, quercetin, which is a well-known inhibitor of RANKL-induced osteoclastogeneis, was used [25]. Quercetin also decreased the number of TRAP-positive multinucleated osteoclasts with the IC_50_ value of 1.9 ± 0.3 µM (Figure S1). We further determined the effects of these two compounds on RANKL-induced expression of osteoclast markers such as c-Src and cathepsin K (CtsK) by western blot analysis. The treatment of BMMs with either AR-3 or AR-5 inhibited RANKL-induced expression of c-Src and CtsK in a concentration-dependent manner (Figure 2C).

Next, we determined the effects of AR-3 and AR-5 on the cell viability of BMMs to determine the possibility that AR-3 and AR-5 inhibited RANKL-induced osteoclastogenesis due to the potentially cytotoxic effects of these compounds on BMMs. However, these compounds did not decrease the cell viability of BMMs, but rather increased the cell viability of BMMs at concentrations utilized in this study (Figure 2D). Collectively, these results suggest that the two 3-hydroxyolean-12-en-27-oic acid derivatives, AR-3 and AR-5, could exert anti-osteoclastogenic effects without decreasing the cell viability of BMMs.

### 2.3. AR-3 and AR-5 Suppress RANKL-Induced Formation of Mature Osteoclasts in Mouse BMMs

**Figure 3:**
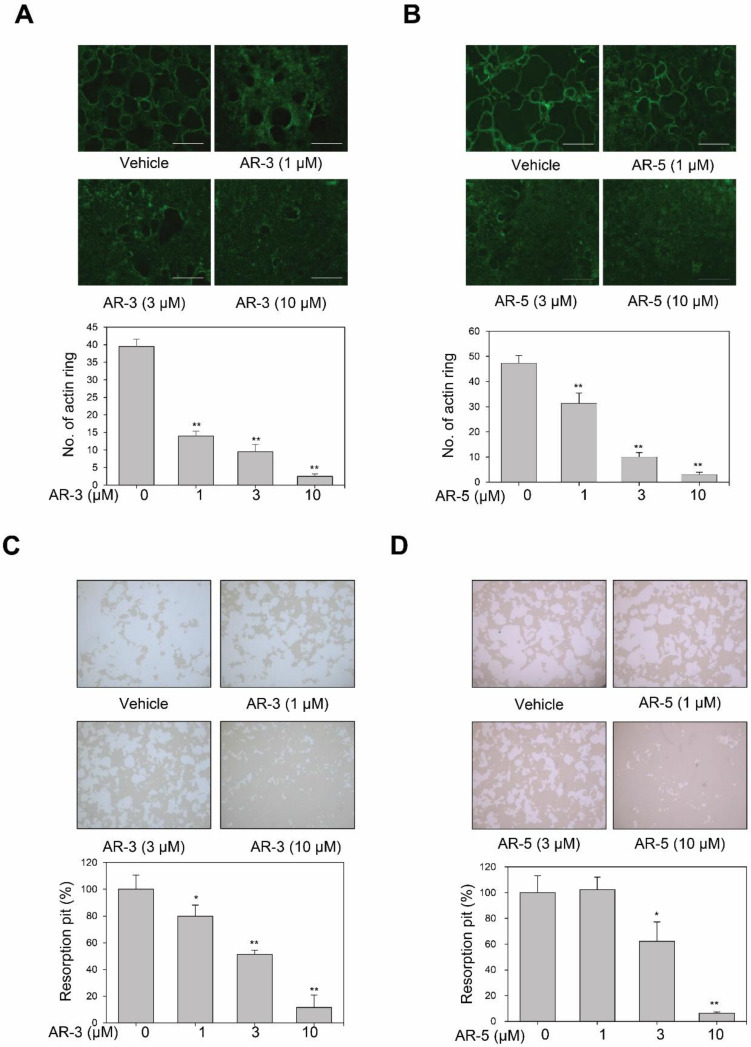
AR-3 and AR-5 inhibit RANKL-induced actin-ring formation and resorption pits in BMMs. (**A**,**B**) BMMs were incubated with M-CSF and RANKL in the presence of indicated concentrations of AR-3 (**A**) or AR-5 (**B**) for seven days. Actin-ring positive osteoclasts were visualized with FITC-conjugated phalloidin (scale bar: 500 μm). Data are presented as the mean ± SE (** *p* < 0.01, versus vehicle-treated control; *n* = 3). (**C**,**D**) BMMs were cultured on Corning OsteoAssay Surface 96 well-plates, and incubated with M-CSF and RANKL in the presence of indicated concentrations of AR-3 (**C**) or AR-5 (**D**) for seven days. The cells were removed and pit area was measured by ImageJ software. Data are presented as the mean ± SE (* *p* < 0.05 and ** *p* < 0.01, versus vehicle-treated control; *n* = 3).

We next determined whether AR-3 and AR-5 could inhibit filamentous actin-ring formation, which is the most obvious characteristic of mature osteoclasts [26]. Stimulation of BMMs with RANKL and M-CSF formed a clear actin-ring structure, as shown by fluorescein isothiocyanate (FITC)-phalloidin staining. However, AR-3 and AR-5 significantly decreased the size and number of actin-ring structures in a concentration-dependent manner (Figure 3A,B). To further confirm whether AR-3 and AR-5 inhibited the RANKL-induced formation of mature osteoclasts, the pit formation assay was performed. These two 3-hydroxyolean-12-en-27-oic acids significantly reduced the density of resorption pits in a concentration-dependent manner (Figure 3C,D). These results suggest that AR-3 and AR-5 could suppress RANKL-induced formation of mature osteoclasts and thereby bone resorption activity.

### 2.4. AR-3 and AR-5 Suppress LPS-Induced Bone Loss in Vivo

**Figure 4:**
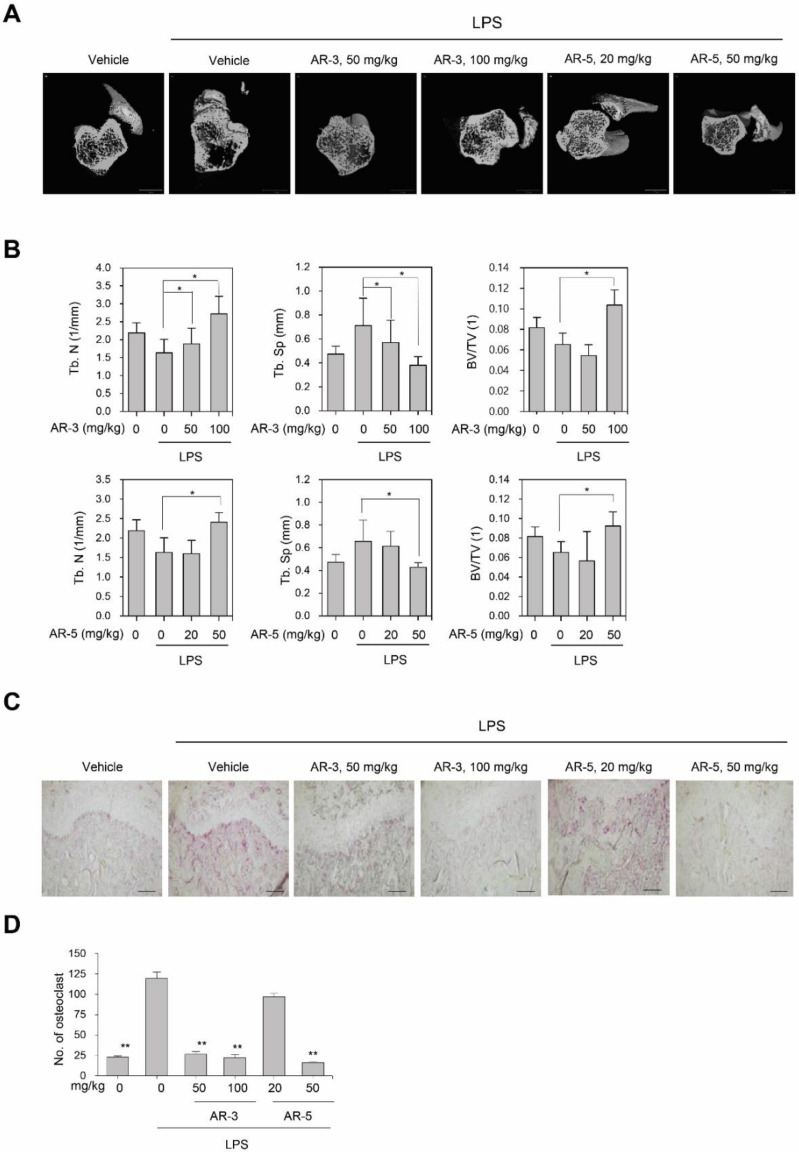
AR-3 and AR-5 suppress LPS-induced bone loss in vivo. (**A**) Mice were sacrificed nine days after the first LPS injection, and three-dimensional images of femurs were obtained using μCT (scale bar: 1 mm). (**B**) Trabecular number (Tb. N), trabecular separation (Tb. Sp), and trabecular bone volume/tissue volume (BS/TV) were analyzed using the CTAn software. *n* = 4 in each group (eight legs). * *p* < 0.05, versus the LPS-treated control. (**C**) Fixed femurs were decalcified and sectioned. Sections were stained with TRAP (200×). Scale bar: 100 µm. (**D**) The number of TRAP-positive osteoclasts was counted. Data are presented as the mean ± SE (** *p* < 0.01, versus the LPS-treated control).

The effects of AR-3 and AR-5 on the suppression of osteoclastogenesis in vivo was assessed using an inflammation-induced bone loss mouse model. The mice were treated with LPS in the presence and absence of AR-3 or AR-5, respectively. After nine days, the femurs were examined by micro-CT. A three-dimensional analysis revealed that the LPS administration caused a marked trabecular bone loss in the femurs. However, oral administration of either AR-3 or AR-5 considerably offered protection from LPS-induced bone loss (Figure 4A). With reference to the micro-CT images, the reduction of trabecular number (Tb.N) and trabecular bone volume/tissue volume (BS/BV) following LPS injection was recovered in AR-3- or AR-5-treated mice (Figure 4B). The LPS-induced increase in trabecular separation (Tb.Sp) was also attenuated by either AR-3- or AR-5 administration in a dose-dependent manner (Figure 4B). Additionally, a histological analysis revealed that the increased number of TRAP-positive osteoclasts induced by LPS was significantly reduced in AR-3- or AR-5-treated mice (Figure 4C,D), further suggesting that both AR-3 and AR-5 inhibit osteoclastogenesis in vivo.

### 2.5. AR-3 and AR-5 Suppress RANKL-Induced NFATc1 Activation

**Figure 5:**
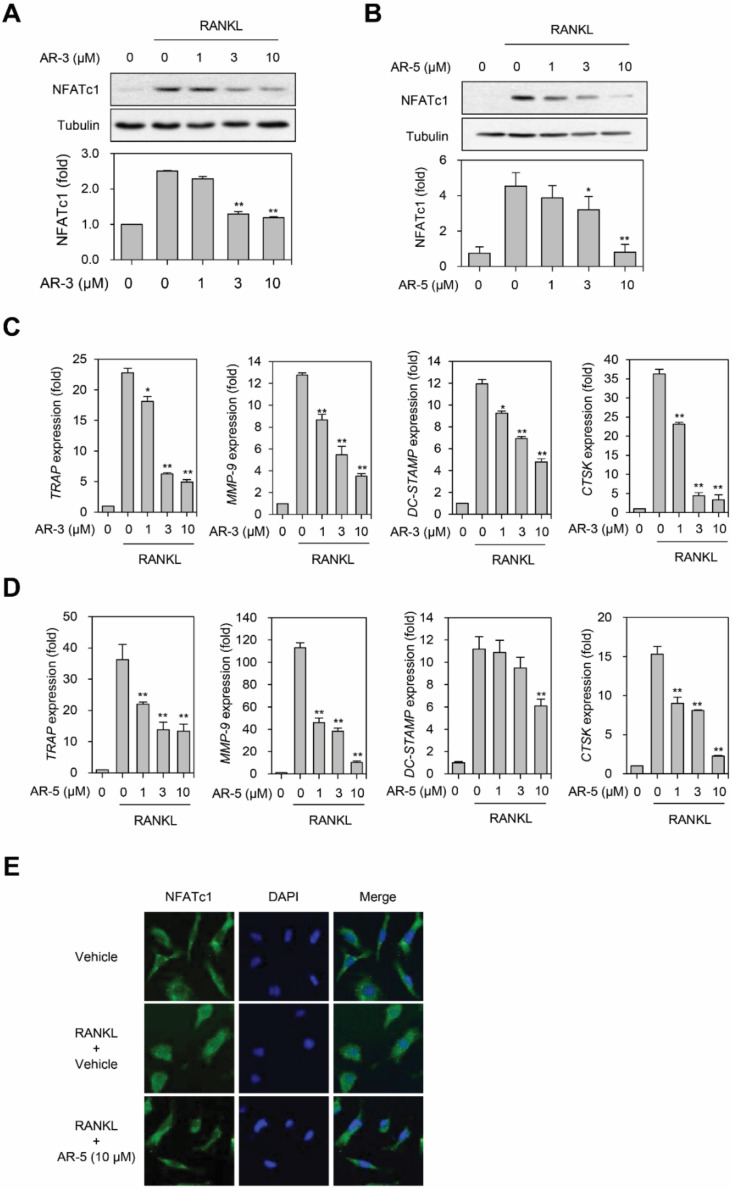
AR-3 and AR-5 inhibit RANKL-induced NFATc1 activation and its target gene expression. (**A**,**B**) RAW264.7 cells were stimulated with RANKL in the presence of the indicated concentrations of AR-3 (**A**) or AR-5 (**B**) for 48 h. The expression level of NFATc1 was determined by western blotting. Graphs represent densitometry analyses normalized to tubulin (* *p* < 0.05 and ** *p* < 0.01, versus vehicle-treated control, *n =* 3). (**C**,**D**) BMMs were incubated with M-CSF and RANKL in the presence of indicated concentrations of AR-3 (**C**) or AR-5 (**D**) for four days. Reverse transcription qPCR analysis was performed to quantify the mRNA expression levels of *TRAP, MMP-9, DC-STAMP*, and *CtsK*. Data are presented as the mean ± SE (* *p* < 0.05 and ** *p* < 0.01, versus the vehicle-treated control; *n* = 5). (**E**) BMMs were stimulated with RANKL and M-CSF in the presence or absence of AR-5 (10 µM) for three days. The cells were fixed and stained with an anti-NFATc1 antibody, followed by an Alexa Fluor 488-conjugated secondary antibody. DAPI was used to stain nuclei.

The activation of NFATc1, a key transcription factor in osteoclastogenesis, directly regulates the induction of numerous osteoclastic genes including CtsK, MMP-9, OSCAR, TRAP, and DC-STAMP, which are involved in the formation of resorption pits during osteoclastogenesis [5,17]. Consequently, we examined whether AR-3 and AR-5 inhibited RANKL-induced expression of NFATc1 and its target genes. AR-3 and AR-5 decreased the RANKL-induced expression level of NFATc1 in a concentration dependent manner (Figure 5A,B). A real-time qPCR analysis revealed that AR-3 and AR-5 significantly decreased the expression level of NFATc1 target genes including *TRAP, MMP-9*, *CtsK*, and *DC-STAMP* in a concentration-dependent manner during RANKL-induced osteoclastogenesis (Figure 5C,D). Immunofluorescence analysis also revealed that AR-5 significantly suppressed RANKL-induced nuclear translocation of NFATc1 (Figure 5E). These results suggest that AR-3 and AR-5 impaired RANKL-induced osteoclastogenesis through inhibiting NFATc1 activation.

### 2.6. AR-5 Inhibits RANKL-Induced Osteoclast Formation at Early and Late Stage of Differentiation, and Migration

**Figure 6:**
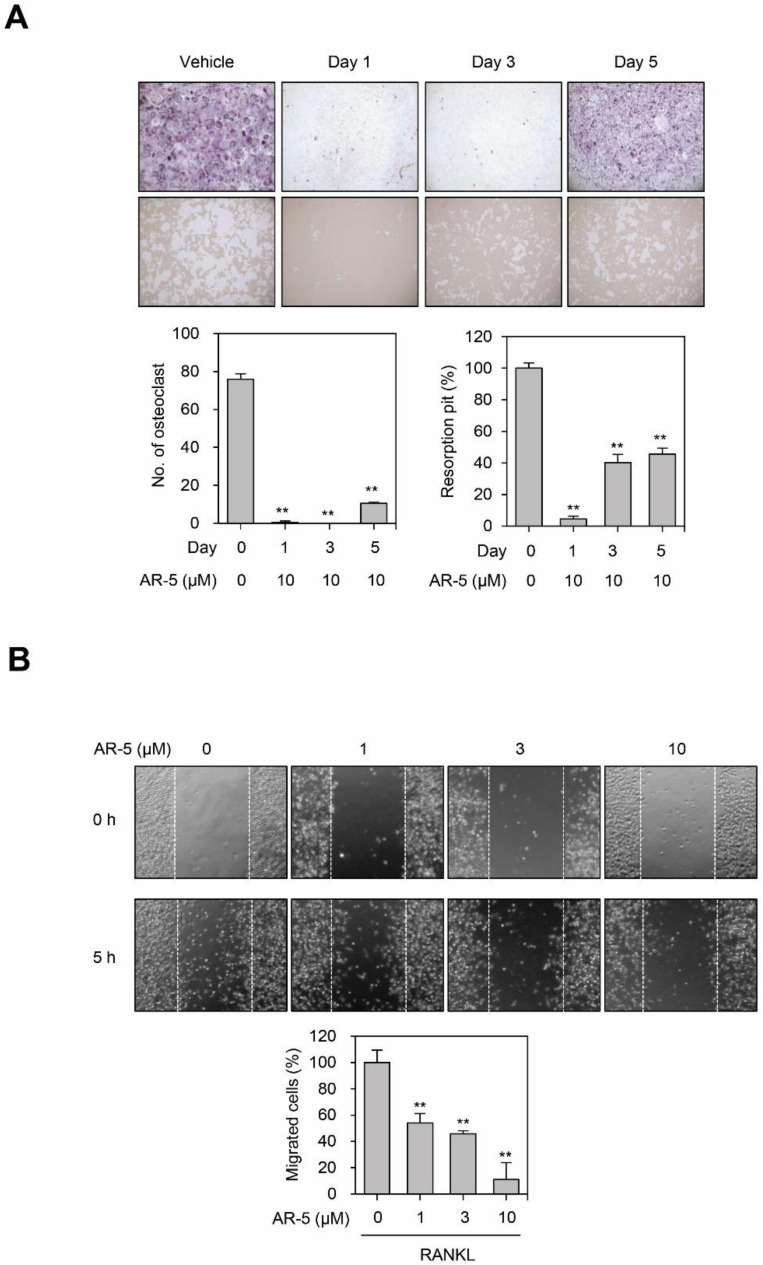
AR-5 inhibits RANKL-induced osteoclast formation at an early and late stage of differentiation. (**A**) BMMs were incubated with M-CSF and RANKL, and then treated with AR-5 (10 µM) at day 1, 3, and 5 after RANKL stimulation. After incubating for seven days, TRAP staining (upper panel) and bone resorption assay (lower panel) were performed. Data are presented as the mean ± SE (** *p* < 0.01, versus vehicle-treated control; *n* = 3). (**B**) Effect of AR-5 on RANKL-induced migration of BMMs. After wounds were made, BMMs were stimulated with M-CSF and RANKL in the presence of the indicated concentration of AR-5. Data are presented as the mean ± SE (** *p* < 0.01, versus vehicle-treated control; *n* = 3).

Since AR-3 and AR-5 inhibited RANKL-induced osteoclastogenesis to a similar extent in vitro and in vivo, we further investigated the mechanisms by which AR-5 inhibits RANKL-induced osteoclast formation. To determine whether AR-5 inhibits an early or late stage of osteoclast formation, we investigated the effects of AR-5 on RANKL-induced osteoclatogenesis by treating at different times of day, from days 1 to 5 after RANKL stimulation (Figure 6A). AR-5 significantly reduced RANKL-induced osteoclast formation and pit formation on day 1, day 3, and day 5 of treatment. This suggests that AR-5 inhibited RANKL-induced osteoclastogenesis at both an early and late stage of osteoclast formation.

Migration of osteoclast precursors is also a critical process during osteoclastogenesis [27,28]. As such, we determined whether AR-5 inhibits RANKL-induced migration of BMMs. The number of migrated cells increased with the stimulation of BMMs with RANKL. However, AR-5 significantly inhibited RANKL-induced migration of BMMs in a concentration-dependent manner (Figure 6B).

### 2.7. AR-5 Inhibits RANKL-Induced Activation of ERK and JNK MAPKs

**Figure 7:**
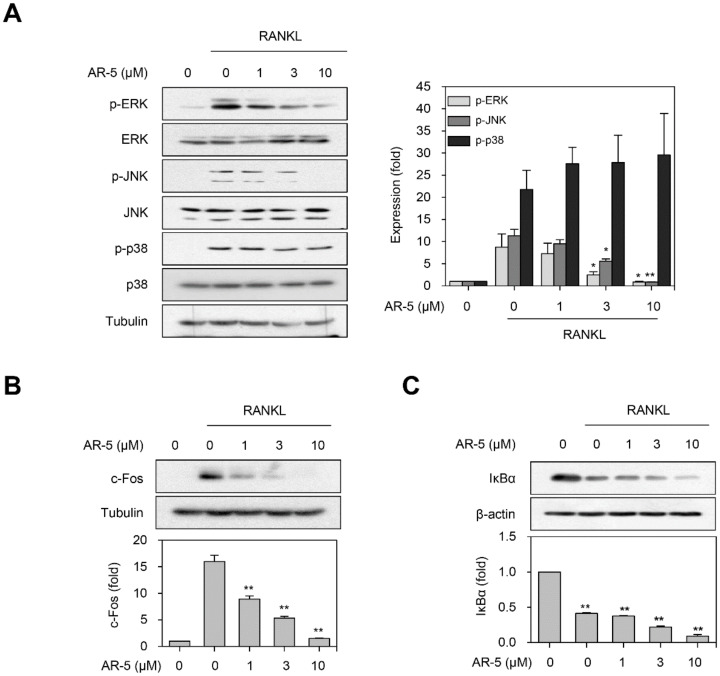
AR-5 inhibits RANKL-induced ERK and JNK phosphorylation, and c-Fos expression. (**A**) RAW264.7 cells were stimulated with RANKL in the presence of indicated concentrations of AR-5 for 10 min. Total lysates were prepared and western blotting was performed to determine the expression level of p-ERK, p-JNK, and p-p38 MAPKs. Densitometry analysis of p-ERK, p-p38, and p-JNK expression (normalized to ERK, p38, and JNK, respectively) expressed as the mean ± SE of three independent experiments (* *p* < 0.05 and ** *p* < 0.01, versus RANKL only-treated control, *n* = 3). (**B**) RAW264.7 were stimulated with RANKL (100 ng/mL) in the presence of the indicated concentrations of AR-5 for 24 h. The expression level of c-Fos was determined by Western blotting. Densitometry analysis of c-Fos expression (normalized to β-actin) expressed as the mean ± SE of three independent experiments (** *p* < 0.01, versus RANKL only-treated control, *n =* 3). (**C**) RAW264.7 cells were stimulated with RANKL (100 ng/mL) in the presence of indicated concentrations of AR-5 for 15 min. The expression level of IκBα was determined by western blotting. Densitometry analysis of IκBα expression (normalized to β-actin) expressed as the mean ± SE of three independent experiments normalized to (** *p* < 0.01, versus RANKL only-treated control, *n* = 3).

We examined the effects of AR-5 on RANKL-induced early signaling events including NF-κB and MAPKs signaling pathways. The phosphorylation levels of ERK, p38, and JNK MAPKs were increased by RANKL treatment. However, pretreatment with AR-5 significantly decreased the phosphorylation levels of ERK and JNK MAPKs in a concentration-dependent manner (Figure 7A). Nonetheless, AR-5 did not suppress RANKL-induced p38 MAPK phosphorylation. Activation of MAPK signaling pathways can lead to induction of the AP-1 transcription factor [16], which is a heterodimer of the c-Jun and c-Fos transcription factors. Thus, we examined whether AR-5 suppressed RANKL-induced c-Fos expression. AR-5 inhibited RANKL-induced c-Fos expression in a concentration-dependent manner (Figure 7B). In contrast, AR-5 did not inhibit RANKL-induced degradation of the IκBα protein (Figure 7C). These results suggest that AR-5 may suppress RANKL-induced osteoclastogenesis by inhibiting the MAPK/AP-1 signaling pathway, but not by NF-κB activation.

## 3. Discussion

Excessive activity of osteoclasts as a result of elevation of RANK/RANKL signaling causes osteolytic bone diseases such as rheumatoid arthritis and osteoporosis. In recent years, there has been an increased interest in the treatment of osteolytic bone diseases using natural products [29,30]. In the current study, we described for the first time that two 3-hydroxyolean-12-en-27-oic acid derivatives, AR-3 and AR-5 isolated from *A. rossi*, inhibited not only RANKL-induced osteoclast formation and function in vitro, but also LPS-induced bone loss in a mouse model.

It is well known that oleanane triterpenes with a carboxyl group at C-28 including 3-hydroxyolean-12-en-28-oic acid derivatives such as oleanolic acid and maslinic acid inhibit RANKL-induced osteoclast formation in vitro and bone loss in animal models [23,24,31]. However, the inhibitory effect of oleanane triterpenes with a carboxyl group at C-27 on osteoclastogenesis has not been investigated. *A. rossii* is a rich source of 12-en-27-oic acid-type triterpenes [31,32,33]. These triterpenes have a variety of biological effects including anticomplementary, cytotoxic, anti-bacterial, anti-cancer, anti-inflammatory, and diacylglycerol aceyltransferase-inhibitory activities [26,32,33,34,35,36,37]. In the current study, we demonstrated that two 3-hydroxyolean-12-en-27-oic acid derivatives, AR-3 and AR-5, suppressed RANKL-induced osteoclast differentiation, actin-ring formation, and resorption pit formation. Additionally, we also showed that these two compounds suppressed RANKL-induced activation of NFATc1 as well as the expression of NFATc1 target genes including TRAP, CtsK, DC-STAMP, and MMP-9, suggesting that AR-3 and AR-5 could exert anti-osteoclastogenic effects by inhibiting RANKL-induced NFATc1 activation. In contrast, olean-12-en-27-oic acid derivatives without a hydroxyl group at C-3 including AR-1, AR-2, AR-4, and AR-5 did not inhibit RANKL-induced osteoclast formation up to 10 µM, suggesting that a hydroxy group at C-2 could be important for exerting the anti-osteoclastogenic effect of olean-12-en-27-oic acids. Thus, olean-12-en-27-oic acid derivatives with a hydroxyl group at C-3 might potentially be lead compounds in the search for antiosteoclastogenic agents and in the development of semisynthetic oleanane-triterpenoid derivatives with anti-osteoclastogenic activities.

The binding of RANKL to its receptor RANK leads to the activation of downstream signaling pathways including ERK, JNK, and p38 MAPKs. The activation of these MAPKs induces the expression of c-Fos, a critical transcription factor for the function of osteoclast precursors [38]. c-Fos is recruited to the NFATc1 promoter to induce autoamplification of NFATc1 during osteoclastogenesis [39,40]. Mice lacking the ERK1 or JNK1 gene exhibit impaired osteoclastogenesis in vivo [41,42] and blockade of JNK signaling inhibits RANKL-induced osteoclast differentiation [43]. In the current study, we illustrated that AR-5 inhibited RANKL-induced activation of ERK and JNK as well as c-Fos expression, suggesting that AR-5 could attenuate RANKL-induced ostyeoclastogenesis through suppression of ERK and JNK activation and subsequent c-Fos expression. However, the detailed mechanism by which AR-5 suppresses RANKL-induced activation of ERK and JNK is yet to be elucidated. NF-κB is one of the important transcription factors that regulate RANKL-induced osteoclastogenesis [44]. Upon stimulation of osteoclast precursors with RANKL, IκB kinase is activated to directly phosphorylate IκBα, leading to release of the NF-κB dimer, which can bind to its target genes in the nucleus [45]. AR-5 did not suppress the RANKL-induced degradation of IκBα, suggesting that AR-5 might not inhibit RANKL-induced NF-κB activation to attenuate RANKL-induced osteoclastogenesis.

In conclusion, we identified two 3-hydroxyolean-12-en-27-oic acid derivatives isolated from *A. rossii*, AR-3 and AR-5, as novel anti-osteoclastogenic compounds in vitro and in vivo. These two compounds are capable of suppressing osteoclast formation and function through impairing RANKL-induced NFATc1 activation. In particular, AR-5 impaired RANKL-induced ERK and JNK MAPKs activation and c-Fos expression. The inhibition of osteoclastogenesis by 3-hydroxyolean-12-en-27-oic acid derivatives may help to provide insight into novel compounds from natural products to be used in developing anti-osteoclastogenic compounds. Consequently, olean-12-en-27-oic acid derivatives with a hydroxyl at C3 could potentially be the lead compounds for the treatment or prevention of osteolytic diseases such as osteoporosis and rheumatoid arthritis.

## 4. Materials and Methods

### 4.1. Chemicals, Reagents, and Antibodies

Antibodies against anti-NFATc1, anti-c-Fos, anti-p38, anti-phospho-p38, anti-JNK, anti-phospho-JNK, anti-ERK, anti-phospho-ERK, and anti-α-tubulin were obtained from Cell Signaling Technology (Danvers, MA, USA). Antibodies against anti-β-actin, anti-CtsK, and anti-c-Src were obtained from Santa Cruz Biotechnology (Santa Cruz, CA, USA). Alexa Fluor 488 goat anti-rabbit secondary antibody and FITC (fluorescein isothiocyanate)-conjugated phalloidin were purchased from Invitrogen (Invitrogen, Carlsbad, CA, USA). M-CSF was obtained from Prospec (East Brunswick, NJ, USA). RANKL was obtained from R&D systems (Minneapolis, MN, USA). DAPI (4′,6-diamidino-2-phenylindole) and quercetin were obtained from Sigma-Aldrich (St. Louis, MO, USA). AR-1, AR-2, AR-3, AR-4, and AR-5 were isolated from *A. rossii* as described previously [33,46,47], and their chemical structure is shown in Figure 1A. These compounds were solubilized in dimethyl sulfoxide and used at final concentrations of less than 0.05% dimethyl sulfoxide.

### 4.2. Cell Culture

RAW264.7 cells were cultured in DMEM (Hyclone, Logan, UT, USA) supplemented with 10% heat-inactivated FBS (Cambrex, Charles City, IA, USA) and penicillin (100 units/mL)-streptomycin (100 μg/mL). Bone marrow cells were isolated from femurs and tibias of six week-old male ICR mice (DBL, Emseong, Chungbuk, Korea) as described previously [27]. Briefly, bone marrow cells were cultured in α-MEM containing 10% FBS, streptomycin (100 μg/mL), penicillin (100 units/mL), and M-CSF (10 ng/mL) overnight at 37 °C in a humidified incubator with 5% CO_2_. Floating cells were cultured for three days with M-CSF (30 ng/mL), and then adherent bone marrow-derived macrophages (BMMs) were used for further experimentation.

### 4.3. In Vitro Osteoclastogenesis Assay

The in vitro osteoclastogenesis assay was performed as described previously [48]. Briefly, BMMs were incubated with the test compounds in the presence of RANKL (100 ng/mL) and M-CSF (30 ng/mL). The compounds and culture media were swapped every two days for six days. RAW264.7 cells were incubated with the test compounds in the presence of RANKL (100 ng/mL). The compounds and culture media were replaced every two days for a total of four days. At the end of incubation, cells were fixed, permeabilized, and then stained with an acid phosphatase leukocyte kit (Sigma-Aldrich). TRAP-positive multinucleated cells (>5 nuclei) were classified as osteoclasts.

### 4.4. Measurement of Cell Viability

Cytotoxic effects of the test compounds on BMMs was assessed using the 3-(4,5-dimethylthiazol-2-yl)-2,5-diphenyl tetrazolium bromide (MTT)-based assay. Briefly, BMMs (5 × 10^5^ cell/mL) were seeded onto 96-well plates and treated with the test compounds in the presence of 30 ng/mL M-CSF. After incubating for 72 h, MTT (0.5 mg/mL) was added and further incubated for 3 h. Insoluble formazan products were dissolved in dimethyl sulfoxide and a microplate reader was used to measure the absorbance at 540 nm.

### 4.5. Bone Resorption Assay

Bone resorption assay was performed as previously described [48]. Briefly, BMMs were seeded at a density of 5 × 10^5^ cell/mL into OsteoAssay Surface 96-well plates (Corning Incorporated, Corning, NY, USA) and stimulated with M-CSF (30 ng/mL) and RANKL (100 ng/mL) in the presence of different concentrations of AR-3 and AR-5. The compound and culture medium were replaced after every two days. After seven days, the cells were completely removed from wells and the plate was thoroughly washed using distilled water. Resorption pits were captured using a model H550L microscope (Nikon Corporation, Tokyo, Japan) and quantified with ImageJ software (Java 1.6.0_20 (64 bit); NIH, Bethesda, MD, USA).

### 4.6. Actin-Ring Formation Assay

BMMs (10^6^ cell/mL) were seeded onto cover glass and stimulated with M-CSF (30 ng/mL) and RANKL (100 ng/mL) in the presence of different concentrations of AR-3 and AR-5 for seven days. The compound and culture medium were replaced every two days. Cells were permeabilized, stained with FITC-phalloidin, and then mounted. Images were captured using an inverted laser scanning confocal microscope (OLYMPUS FV1000, Shinjuku, Tokyo, Japan) equipped with an external argon laser, HeNe laser Green, and HeNe laser Red. Images were captured at colony midsection using a UPLSAPO 60X NA1.35 oil immersion objective (OLYMPUS).

### 4.7. Migration Assay

For the wound healing migration assay, BMMs were cultured in 6-well plates and a linear wound was created in the confluent monolayer by scratching using a 10 µL micropipette tip. After washing three times with media to remove unattached cells, the cells were then stimulated with M-CSF (30 ng/mL) and RANKL (100 ng/mL) in the presence of different concentrations of AR-5. After five hours of incubation, cells were fixed and the wound edge closure was measured with a microscope and the number of migrated cells was standardized to the vehicle treated control.

### 4.8. Western Blot Analysis

For the preparation of whole cell lysates, the cells were lysed in a lysis buffer (50 mM Tris-HCl [pH 7.5], 1% Nonidet P-40, 1 mM EDTA, and 150 mM NaCl) in the presence of a protease inhibitor cocktail (Sigma-Aldrich). The lysates were resolved by sodium dodecyl sulfate-polyacrylamide gel electrophoresis, followed by western blot analysis. Western blots were incubated overnight with the indicated primary antibodies (1:1000 dilution), and anti-rabbit or anti-mouse secondary antibodies conjugated to horseradish peroxidase (1:2000 dilution) were used to visualize signals using an enhanced chemiluminescence system (ThermoFisher Scientific, Rockford, IL, USA). The band intensity was quantified using ImageJ software (NIH, Bethesda, MD, USA).

### 4.9. Immunofluorescence and Confocal Microscopy

Cells were rinsed once in PBS, fixed in fresh 4% paraformaldehyde for 20 min at room temperature, then permeabilized in 0.5% TritonX-100. Nonspecific sites were blocked by incubating with PBS containing 1% goat serum before incubating the cells with an antibody against anti-NFATc1 (1:200 dilution). After washing four times with PBS, the cells were incubated with Alexa Fluor 488 goat anti-rabbit secondary antibody (1:250 dilution) for 4 h at room temperature, washed, stained with DAPI, and mounted. Confocal images were acquired using an OLYMPUS FV1000 inverted laser scanning confocal microscope equipped with an external argon laser, HeNe laser Green, and HeNe laser Red. Images were captured at colony midsection using a UPLSAPO 60X NA1.35 oil immersion objective (OLYMPUS).

### 4.10. Reverse Transcription qPCR Analysis

The cells were collected and total RNA was isolated using an RNeasy Mini Kit according to the manufacturer’s protocol (Qiagen, Valencia, CA, USA). First-strand cDNA was prepared from 1 μg of the total RNA. Real-time qPCR analysis was performed using TOPreal qPCR 2X PreMIX (SYBR Green; Enzynomics, Inc., Daejeon, Korea) and a Rotor-Gene Q real-time PCR cycler (Qiagen). The primers used included: *CtsK*, 5′-GAC ACC CAG TGG GAG CTA TG-3′ (sense) and 5′-AGA GGC CTC CAG GTT ATG GG-3′ (antisense); *TRAP*, 5′-ACT TGC GAC CAT TGT TAG CC-3′ (sense) and 5′-TTC GTT GAT GTC GCA CAG AG-3′ (antisense); *MMP-9*, 5′-TGG GCA AGC AGT ACT CTT CC-3′ (sense) and 5′-AAC AGG CTG TAC CCT TGG TC-3′ (antisence); *β-actin*, 5′-GGG AAA TCG TGC GTG ACA TCA AAG-3′ (sense), and 5′-AAC CGC TCC TTG CCA ATA GT-3′ (antisense). The primers for *DC-STAMP* were purchased from Origene (Rockville, MD, USA). PCR conditions were: 95 °C for 10 min, followed by 40 cycles at 95 °C for 10 s, 60 °C for 15 s, and 72 °C for 20 s. All reactions were performed in triplicate and β-actin was utilized as an internal control. The 2^−ΔΔCq^ method was used to determine the relative gene expression.

### 4.11. Animal Experiments

All experimental protocols were approved by the Institutional Animal Care and Use Committee (IACUC) of Kangwon National University (IACUC approval no. KW-180119-3). Six week-old ICR-mice were randomly divided into control vehicle-treated, vehicle + LPS-treated, AR-3 + LPS-treated, and AR-5 + LPS-treated groups (*n* = 4/group). The mice were orally administrated with AR-3 (50 and 100 mg/kg, dissolved in corn oil), AR-5 (20 and 50 mg/kg, dissolved in corn oil), or the control vehicle (corn oil) for 1 h before the first administration of LPS (5 mg/kg) via intraperitoneal injection and then every other day for eight days. LPS was intraperitoneally injected on days 2 and 6. The mice were dissected on day 9. The femurs of the mice were collected and fixed in 4% paraformaldehyde. Intact left femoral metaphysic regions of each mouse were evaluated by high-resolution micro-CT analysis using an NFR-Polaris-S160 apparatus (Nanofocus Ray; Korea Basic Science Institute Chuncheon Center) with a current of 90 μA, a source voltage of 45 kVp, and an isotropic resolution of 7 μm. Femoral scans were performed over 2 mm from the growth plate, with a total of 350 sections per scan. After three-dimensional reconstruction, trabecular number (Tb.N), bone mineral density (BMD), trabecular separation (Tb.Sp), and bone surface/bone volume (BS/BV) were examined with quantitative analyses using INFINITT-Xelis software (version 1.7; INFINITT Healthcare Co. Ltd., Seoul, Korea). For histological analyses, the femurs were fixed in 4% paraformaldehyde (Sigma) for a day, decalcified in 12% EDTA for three weeks, and embedded in paraffin. Sections of 5 μm thickness were prepared and stained using TRAP in order to examine the osteoclast formation.

### 4.12. Statistical Analysis

Data were expressed as mean ± standard error (SE). Statistical significance was assessed by one-way analysis of variance and Tukey’s test in order to examine the differences between the two groups using SPSS version 14.0 (SPNN Inc., Chicago, IL, USA). *p* values of less than 0.05 were considered statistically significant.

## Supplementary Materials

The following are available online at https://www.mdpi.com/1422-0067/21/15/5240/s1. Figure S1: Effect of quercetin on RANKL-induced osteoclast formation.

## Abbreviations

AR-1	3α,23-isopropylidenedioxyolean-12-en-27-oic acid
AR-2	3-oxoolean-12-en-27-oic acid
AR-3	3α-hydroxyolean-12-en-27-oic acid
AR-4	23-hydroxy-3-oxoolean-12-en-27-oic acid
AR-5	aceriphyllic acid A
BMM	bone marrow-derived macrophage
BS/BV	bone surface/bone volume
CT	Computed Tomography
CtsK	cathepsin K
DAPI	4′,6-diamidino-2-phenylindole
DC-STAMP	dendritic cell–specific transmembrane protein
ERK	extracellular signal-regulated kinase
FITC	fluorescein isothiocyanate
JNK	c-Jun N-terminal kinase
LPS	lipopolysaccharide
MAPK	mitogen-activated protein kinase
M-CSF	macrophage colony-stimulating factor
MMP-9	matrix metallopeptidase 9
MTT	3-(4,5-dimethylthiazol-2-yl)-2,5-diphenyl tetrazolium bromide
NFATc1	nuclear factor of activated T cells cytoplasmic 1
NF-κB	nuclear factor-κB
OSCAR	osteoclast-associated receptor
qPCR	quantitative polymerase chain reaction
RANK	receptor activator of nuclear factor-κB
RANKL	receptor activator of nuclear factor-κB ligand
Tb.N	trabecular number
Tb.Sp	trabecular separation
TARF6	TNF-receptor-associated factor 6
TRAP	tartarate-resistant acid phosphatase
