# Supplementary material for: 3-Hydroxyolean-12-en-27-oic Acids Inhibit RANKL-Induced Osteoclastogenesis in Vitro and Inflammation-Induced Bone Loss in Vivo
Source: Int J Mol Sci. 2020 Jul 23;21(15):5240. doi: 10.3390/ijms21155240 (PMC7432734; doi:10.3390/ijms21155240)
Supplement: Supplementary file 1 [file ijms-21-05240-s001.pdf]

Supplementary materials

Anti-osteoclastogenic activities of 3-hydroxyolean-12-en-27-oic acid derivatives *in vitro* and *in vivo*

Wonyoung Seo [1,2,†], Suhyun Lee [1,2, †], Phuong Thao Tran [1,2], Thi Quynh-Mai Ngo [3], Okwha Kim [1,2], Thanh Huong Le [4], Nguyen Hai Dang [4], Cheol Hwangbo [5], Byung Sun Min [3] and Jeong-Hyung Lee[1,2,*]

Figure S1

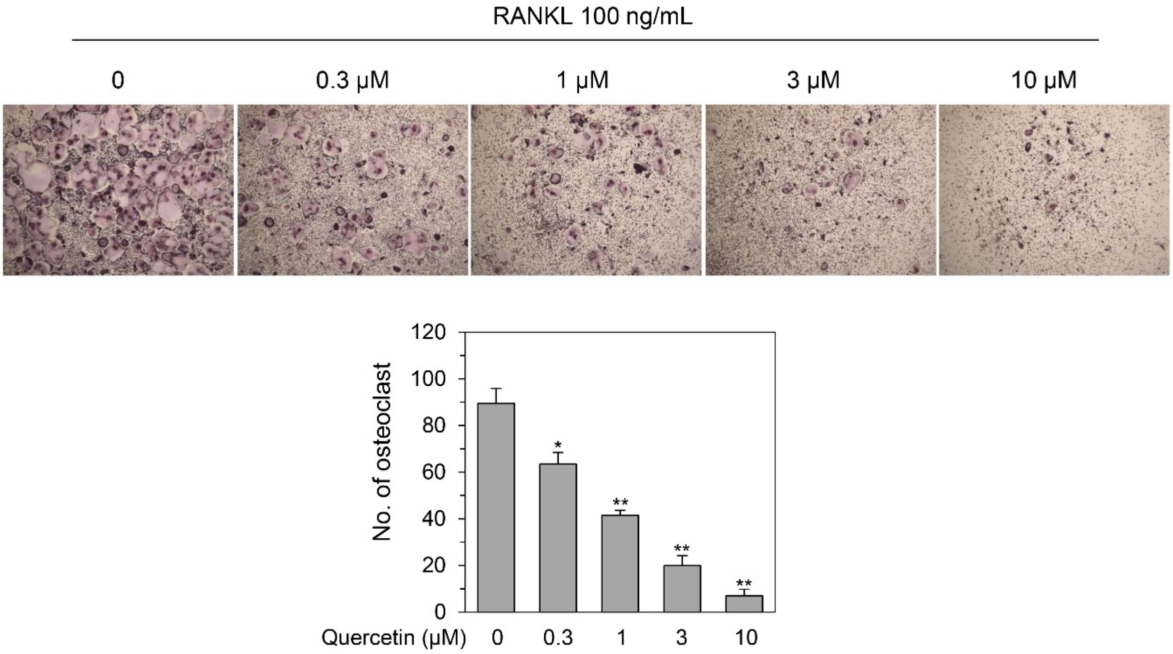

Figure S1. Effect of quercetin on RANKL-induced osteoclast formation. BMMs were treated with the indicated concentrations of quercetin**,** and then stimulated with M-CSF (30 ng/mL) and RANKL (100 ng/mL) for 7 days. Cells were fixed and stained for TRAP. The number of TRAP-positive osteoclasts (>5 nuclei) was determined following image capture (magnification, 40×). Data are presented as the mean ± SE (***P*<0.01, versus vehicle-treated control; n=3).
